# Control of Snail1 protein stability by post-translational modifications: the basis for a complex regulation of Snail1 function

**DOI:** 10.7150/ijbs.108903

**Published:** 2025-04-28

**Authors:** Antonio García de Herreros

**Affiliations:** 1 Programa de Recerca en Càncer, Hospital del Mar Research Institute (IMIM), Unidad Asociada al CSIC, Barcelona, Spain.; 2 Departament de Medicina i Ciències de la Vida, Universitat Pompeu Fabra, Barcelona, Spain.

**Keywords:** Snail1, ubiquitination, phosphorylation, Snail1 structure

## Abstract

Snail1 transcriptional factor is essential for the epithelial to mesenchymal transition and for the acquisition by tumor cells of properties associated to this transition, such as increased invasion and chemoresistance. Snail1 function is mainly controlled post-translationally, through different modifications that directly or indirectly control Snail1 protein stability. In this review I describe these modifications, the enzymes that produce them and their relevance for Snail1 function, focusing particularly in polyubiquitination and phosphorylation. I also propose several explanations for the divergent effects of some of these modifications, since the phosphorylation of some residues have been reported to both promote and decrease Snail1 stability. Moreover, I discuss the possible causes of the observed Snail1 promiscuity in the interaction with the many factors involved in its regulation, on the basis of the *in silico* proposed Snail1 structure.

## 1. Introduction

Snail1 is a transcriptional factor with a key role in the control of the epithelial-to-mesenchymal transition (EMT). As a consequence, Snail1 expression in tumor cells provides features associated to this transition such as increased invasion and chemoresistance 1. In EMT, Snail1 acts as a transcriptional repressor, binding directly to the promoter and blocking the expression of key epithelial genes, such as CDH1, and also activating the expression of mesenchymal genes; in this case, through a more indirect interaction with these genes' promoters 2. Snail1 also leads the expression of other transcriptional factors controlling EMT, such as Zeb1/2, Snail2 and Twist, factors that are collectively known as EMT-TFs 1, 3. In accordance with its role in EMT, Snail1 expression is rapidly induced by growth factors and cytokines promoting this transition, such as EGF, HGF, TGFβ, IL6, endothelin-1, Wnt5a and TNFα, and also by other conditions causing cellular stress, such as hypoxia, ultraviolet and gamma irradiation or chemotherapeutic drugs 4-14. Although some of these conditions also stimulate Snai1 gene transcription, Snail1 up-regulation is mainly produced by an increase in protein stability dependent on different mechanisms, involving phosphorylation, reduced Snail1 E3 ligase expression or elevated Snail1 deubiquitinases. Different reviews have been published on the Snail1 function in EMT, chemo-resistance and even acquisition of cancer stem cell properties 2, 15-16. In this article I focus in the specific regulation of this factor by post-translational modifications that control its function, affecting subcellular localization and protein stability. I extensively analyze these different modifications that, in some cases produce contrary actions on Snail1 function, and discuss their relation with the proposed Snail1 structure.

## 2. Snail1 ubiquitination

**Figure 1:**
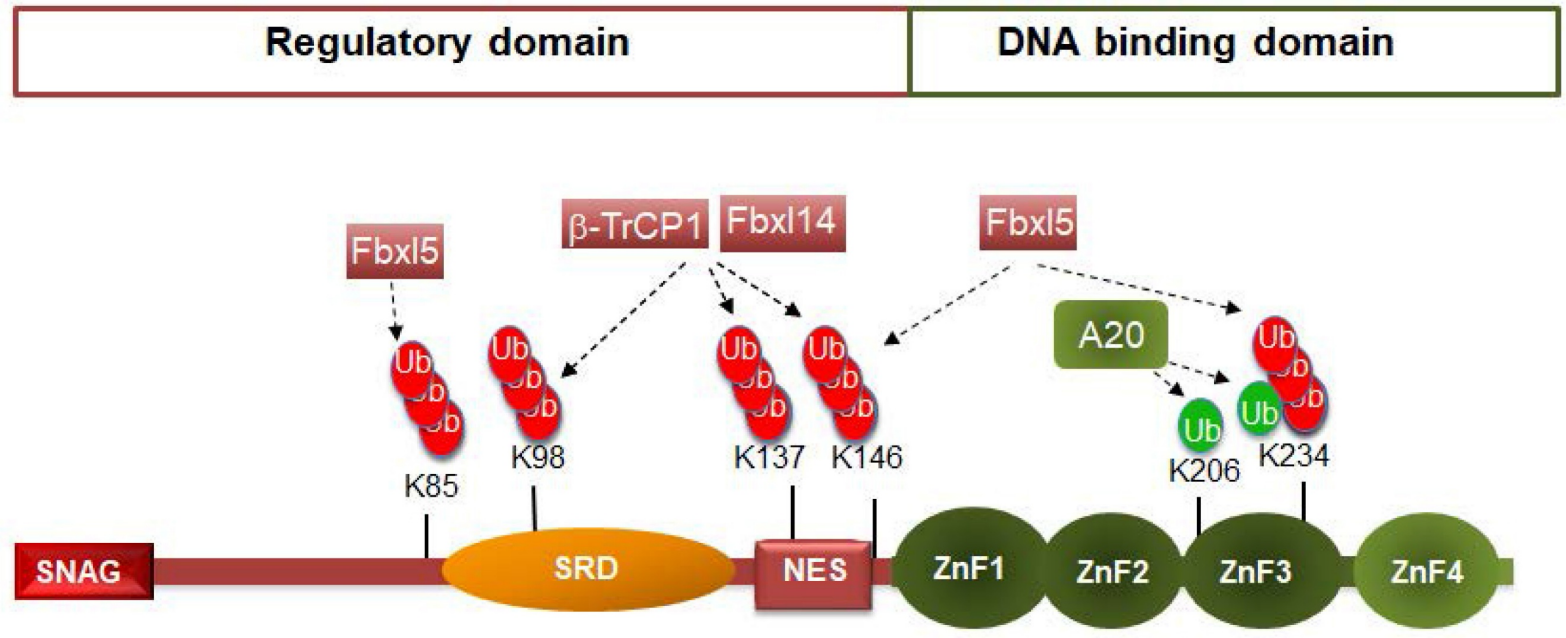
** Snail1 ubiquitination by E3 ubiquitin ligases.** The figure shows a diagram of murine Snail1 protein depicting the N-terminal regulatory domain, comprising the SNAG sequence, Ser-rich domain (SRD) and the NES element; and the C-terminal DNA-binding domain, with the four Zinc fingers. Ubiquitination of the amino acids are presented in green or red if they activate or inhibit (respectively) Snail1 function. The enzymes that modify these residues are shown. Although not reported, these amino acids are probably modified by many other E3 ligases.

Snail1 protein is composed by 264 amino acids structured in two well-defined domains (see Figure 1). The C-terminal domain binds directly to DNA, to a consensus 5'-CACCTG-3' sequence (or inverse, 5'-CAGGTG-3') present in the repressed epithelial genes, such as CDH1. The interaction with the DNA takes place through three Cys2-His2 (C2H2) zinc fingers (ZnF) with the standard consensus Cys-X2,4-Cys-X12-His-X3,5-His and a fourth ZnF with a Cys instead of the last His (C2HC). This last ZnF is less relevant for DNA binding 17 and it is likely involved in the interaction with other proteins, as often happens with C2HC ZnFs 18. The C-terminal domain also includes the nuclear localization sequence 19-20 and is the element involved in the Snail1 interaction with factors related to its function as transcriptional activator, such as NFkB or the GATA zinc finger protein p66β 2. In contrast, the regulatory domain presents a short sequence in the N-terminus, the SNAG domain, required for binding to co-repressors. Other relevant regions in the N-terminal domain are the Ser-rich subdomain (SRD) (amino acids 90-120) and the nuclear-export sequence (NES) (amino acids 132-144) 21 (Figure 1). Although they display the same general organization in two domains, other members of the SNAIL family exhibit relevant differences in the regulatory domain. For instance, Snail2 (Slug) does not contain the SRD or the NES but a specific 28 amino-acid region, called the SLUG domain that is required for CDH1 repression 22. Although Snail2 is also phosphorylated (for instance, see 23), the post-translational modifications of this factor have been less investigated, probably reflecting the less prominent role that Snail2 has in EMT.

Although Snail1 gene expression is regulated at multiple levels 3, the control of Snail1 protein stability is especially relevant, similar to what happens with other key transcriptional factors, such as c-myc, p53 or β-catenin. In most cells, Snail1 is a short-lived protein (with a half-life of about 25 min) and is rapidly ubiquitinated and degraded by the 26S proteasome 24. Actually, Snail1 is ubiquitinated by seventeen different E3 ubiquitin ligases, most of the Cullin-RING type (see Table 1). The Lys residues modified by these ubiquitin ligases have been identified only in a few cases (Figure 1), but they seem to be preferentially located in the SRD and NES subdomains.

The action of so many E3 ligases on Snail1 suggests a highly redundant mechanism of protein degradation to maintain Snail1 levels very low under non-pathological conditions. However, some relevant points have been very little studied; for instance, the coordination of action of these E3 ligases. It is possible that some Snail1 E3 ligases work just initiating the process of ubiquitination and other enzymes more active on Snail1 are in charge of extending polyubiquitinated Lys48-mediated chains. At this regard, Snail1 down-regulation caused by FBXL5, an E3 ligase present in the nucleus, as Snail1, is sensitive to the inhibition of Snail1 export 25. These results have been explained proposing that FBXL5 ubiquitinates Snail1 in the nucleus to release it from the DNA and to enable the accessibility of the NES sequence to the nuclear export complex; in the cytosol, Snail1 is degraded after the polyubiquitination is completed by FBXL14 or βTrCP1, two enzymes present in this compartment that exhibit high activity on Snail1 25.

Many Snail1 E3 ligases bind to a phospho-degron. The best example of a phosphorylation-dependent interaction is that of βTrCP1 that requires the previous phosphorylation by GSK3β of Ser96 and Ser100 located in the SRD (Figure 1) 26. Phosphorylation by the protein kinase D1 (PKD1) or AMP-activated protein kinase (AMPK) of Snail1 Ser11 in the SNAG domain is also required for Snail1 degradation by FBXO11 27-28; however, this phosphorylation-dependent interaction has been discussed by other authors 29. Snail1 is also degraded by FBXW7 30-31. In this case, the requisite of Snail1 phosphorylation has not been assessed, although FBXW7 binds phospho-degrons in other substrates such as ZEB2 32-33. Three other Snail1 E3 ligases, FBXO31, FBXO22 and SPSB3 also exhibit phosphorylation-dependence when inducing Snail1 proteolysis 34-36. However, in these cases it is not totally evident that this is consequence of the generation of a phospho-degron on Snail1 since it might be related to the stimulation of Snail1 nuclear export caused by GSK3 phosphorylation (see below). Since most of E3 ligases are present in the cytosol and nuclear export is dependent on Snail1 phosphorylation, the inhibition of Snail1 degradation in Snail1 phosphorylation-deficient mutants might be consequence of a deficient nuclear export and not because the requirement of a phospho-degron.

Snail1 *in vitro* binding to other ligases is independent on phosphorylation; for instance, that to FBXL14 9. FBXL14 and βTrCP1 are probably the most active Snail1 E3 ligases and redundantly modify the same group of Snail1 lysines (Lys98, 137 and 146) 9. Although *in vitro* binding of FBXL14 to Snail1 is not phosphorylation-dependent, *in vivo* it might be, since FBXL14 is a cytosolic protein and Snail export to the cytosol is stimulated by GSK3β-dependent Snail1 phosphorylation. Moreover, Snail1 degradation by FBXL14 is stimulated by LKB1, that interacts with both proteins 37.

FBXL14 has a central role in EMT since it also targets other EMT-TFs such as Snail2, Twist1 and Zeb2 38. This common down-regulation of EMT-TFs is also shared by other Snail1 E3 ligases: FBXO45, that also regulates Snail2, Zeb1/2 and Twist1 39, FBXW7, that targets Zeb2 33, and TRIM1, that polyubiquitinates Snail2 40.

Another interesting issue is the autophagic degradation of Snail1 in the lysosome. It has been reported that Snail1 is also degraded through selective autophagy 41 although the precise mechanism remains to be stablished. Interestingly, Snail1 is targeted by two members of the TRIM family that work as E3 ubiquitin ligases: TRIM21 and TRIM50 40, 42. TRIM proteins are E3 ligases known to regulate autophagy 43; in some cases, through Lys63-mediated polyubiquitination 44. Moreover, TRIM21 promotes autophagic degradation of other transcriptional factors (IRF3 and c-myc) 45-46. Alike these factors, it is possible that Snail1 undergoes Lys63-polyubiquination by TRIM50 or TRIM21 and subsequent lysosomal degradation. It is also possible that Snail1 interaction with these E3 ligases is mediated by HSP70 (HCS70), since this chaperone promotes Snail1 lysosomal targeting 47.

Snail1 polyubiquitination is not always related to degradation, since two E3 ubiquitin ligases acting on Snail1 increase its protein stability (Table 1). Pellino-1 promotes Snail1 Lys63-mediated polyubiquitination and increases Snail1 half-life 48. A20 multi-monoubiquitinates and stabilizes Snail1 49. According to these authors, monoubiquitination of Lys 206, 234 and 235 is particularly relevant for stabilization. However, Lys235 is not present in the murine Snail1 and Lys234 is also targeted by FBXL5 and affects DNA binding 25. Therefore, it is likely that Lys206 monoubiquitination facilitates Snail1 interaction with some nuclear structure, preventing its export and degradation.

Snail1 ubiquitination can be reversed by deubiquitinating enzymes (deubiquitinases, or DUBs). Alike Snail1 E3 ligases, many DUBs acting on Snail1 has been described (Table 2), suggesting again that Snail1 protein stability is finely regulated. Of note that several of these DUBs are induced by factors that considerably up-regulate Snail1 expression: for instance, DUB3 (also known as USP17L2), by IL-6; USP27X, by TGFβ; and USP47, by hypoxia 12-13, 50-51. The relevance of these DUBs in Snail1 expression is stressed by results as those obtained in the broadly studied model of EMT consisting in NMuMG cells treated with TGFβ: USP27X activation by this cytokine is totally required for the expression of Snail1 13.

Snail1 DUBs are present in the cytosol since polyubiquitination takes place mostly in this compartment. However, some DUBs exhibit specific localizations. This is the case of USP36, present in the nucleolus where binds and stabilizes Snail1 52. This is related to the role of Snail1 promoting ribosomal RNA synthesis and ribosome biogenesis, required for the completion of EMT 53. How Snail1 is targeted to this nuclear sub-compartment remains to be investigated.

Alike Snail1 E3 ligases, some Snail1 DUBs also deubiquitinate other EMT-TFs. This is the case for DUB3, that also works on Snail2 and Twist 54, and USP10 and USP36, capable to bind and stabilize Snail2 55-56.

Since Snail1 expression provides tumor cells with a high resistance to chemotherapy 1, depletion of many Snail1 DUBs has been associated to chemosensitivity. However, only two Snail1 DUBs exhibit an increased function upon exposition to drugs: USP1, that is phosphorylated by ATM and ATR upon cisplatin treatment enhancing its binding to Snail1 57, and USP29, that is upregulated by taxol and doxorubicin 58. USP29 presents another interesting feature since it stabilizes Snail1 not only through its DUB activity but also enhancing Snail1 interaction with the small C-terminal phosphatase 1 (SCP1) 59. SCP1 promotes Snail1 dephosphorylation 60 and prevents Snail1 degradation by the E3 ligases that recognize phospho-degrons or by those present in the cytosol, since phosphorylation also controls Snail1 nuclear export (see below).

As an additional point stressing the relevance of the control of Snail1 stability, polyubiquitination is also regulated by different proteins that interfere with the function of Snail1 E3 ubiquitin ligases. For instance, the up-regulation in Snail1 caused by TNFα is partially mediated by the NFκB-induced COP9 signalosome 2 protein (CSN2), which blocks Snail1 ubiquitination by βTrCP1 and likely by other E3-ligases since it inhibits the activity of E3 ligases requiring cullin 61. In the same direction, EDAR-associated death domain protein (EDARADD) stabilizes Snail1 preventing degradation by TRIM21 by a multiple mechanism involving interaction with and degradation of this E3 ligase and also blocking its transcription 62. Other proteins interacting with Snail1, such as Elongator protein 3 (Elp3), flotillin and ERK3 also stabilize Snail1 preventing the action of E3 ligases on this protein 63-65. In contrast, the regulation of Snail1 DUBs has been less studied and has been just associated to their expression. Only the activity of USP5 is dependent on the previous phosphorylation of Snail1 by MSK1, that enhances the binding of this DUB and increases Snail1 stability 66. On the contrary, nucleoredoxin (NXN) facilitates Snail1 destabilization though its binding to DUB3 what inhibits the action of this DUB on Snail1 67.

Therefore, Snail1 is polyubiquitylated and deubiquitinated by multiple E3 ligases and DUBs that finely regulate Snail1 protein expression in different cells at under different stimuli. The action of these enzymes on Snail1 is controlled through Snail1 protein phosphorylation, that modulates their binding and accessibility to Snail1 protein and also by proteins that interact with Snail1 and preclude its polyubiquitination.

## 3. Snail1 phosphorylation

**Figure 2:**
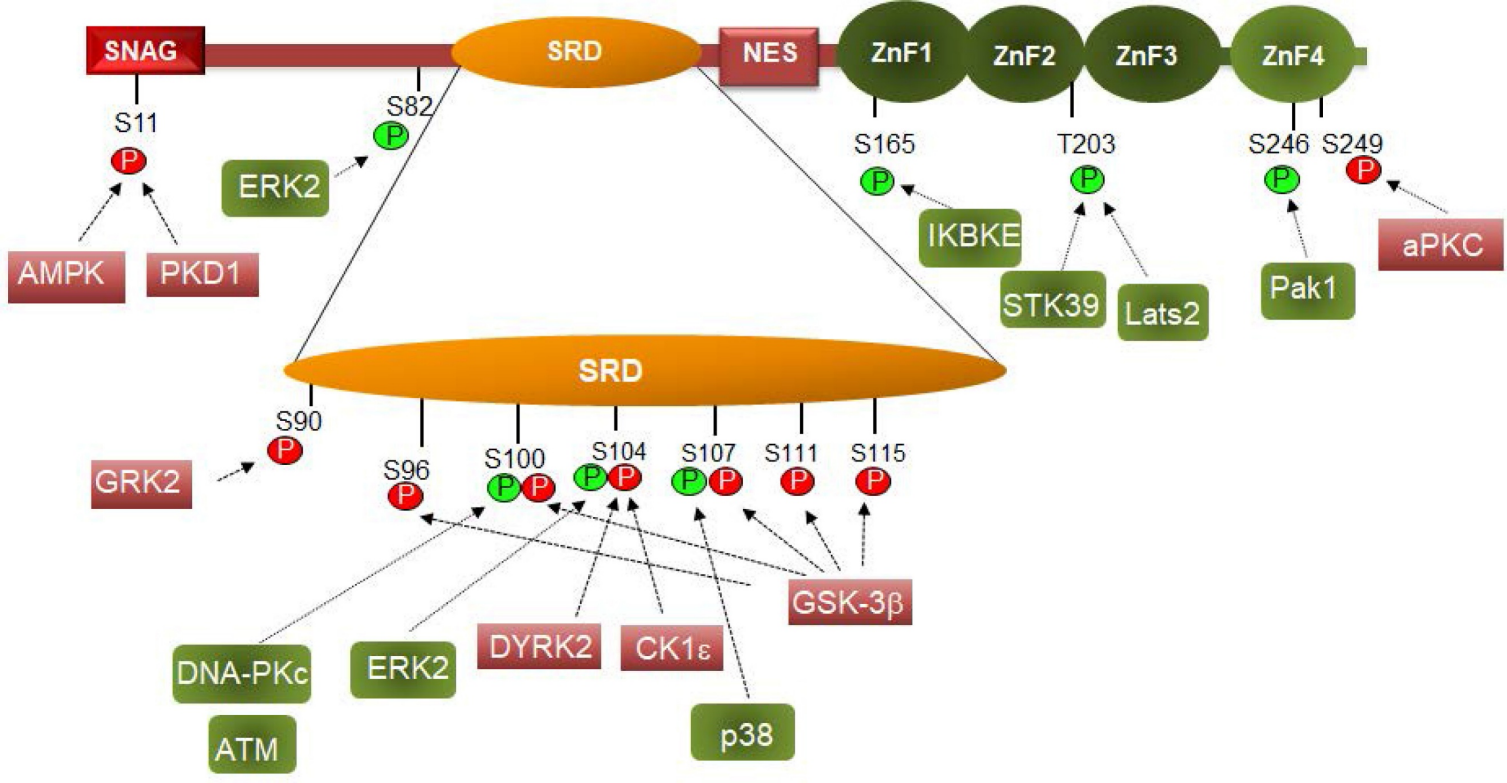
** Phosphorylation controls Snail1 function**. Phosphorylation of the indicated amino acids are depicted in green or red if they activate or inhibit (respectively) Snail1 function. Please notice that phosphorylation of some residues can promote a positive or negative effect on Snail1 function depending on the subcellular localization. The protein kinases that modify these residues are shown. Only those sites that are modified by an identified protein kinase are presented here.

**Figure 3:**
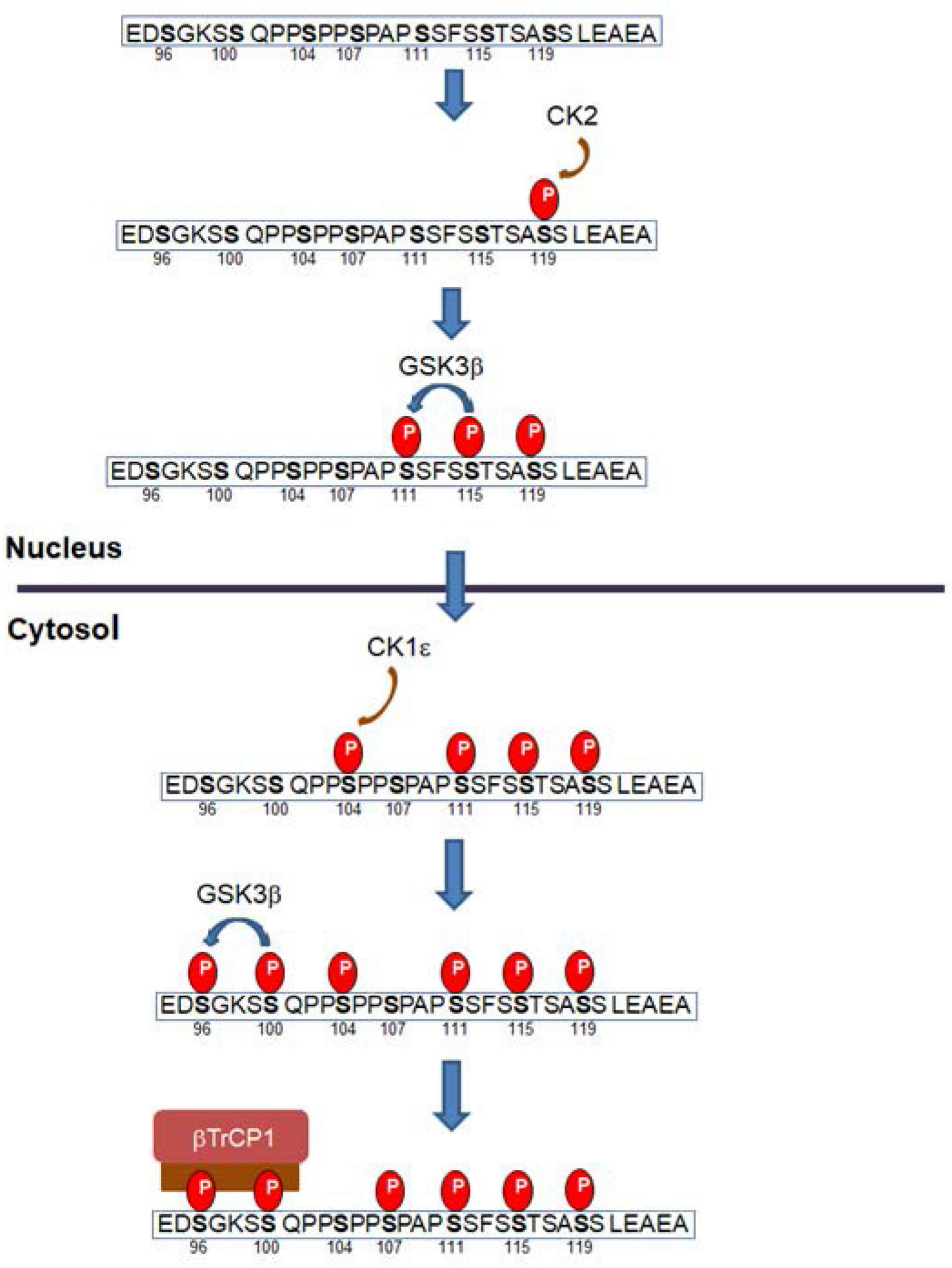
** Progressive Snail1 phosphorylation promotes Snail1 nuclear export and degradation**. According to this model, Snail1 is initially phosphorylated at Ser119 in the nucleus by CK2. This primes Snail1 for the successive phosphorylation by GSK3β of Ser115 and Ser111. Phosphorylation of these sites exposes the NES present in amino acids 138 and 146 and promotes Snail1 nuclear export. At the cytosol, CK1ε phosphorylates Snail1 Ser104; this triggers the phosphorylation of Ser100 and Ser96 by GSK3β, generating a phospho-degron and promoting binding of βTrCp1 E3 ligase and subsequent Snail1 degradation. Of note that Snail1 export from the nucleus to the cytosol can promote also its polyubiquitination by other E3 ligases resident in this compartment, such as Fbxl14, that does not require Ser96 or Ser100 phosphorylation. Only the SRD domain of Snail1 is depicted in this figure; the sequence corresponds to the murine one; the human sequence is identical except a Val-Ala change in position 118.

Snail1 protein is modified by different Ser and Thr protein kinases: thirteen different residues have been reported to be phosphorylated (Figure 2). Most of these modifications take place in the Ser-rich sequence (SRD) (amino acids 90-120). GSK3β is the protein kinase displaying the highest activity on this domain. Two sequences are phosphorylated in the SRD, related to two different actions of GSK3β on Snail1. First, this protein kinase phosphorylates Ser residues 107, 111 and 115, uncovering a NES placed at amino acids 132-144 and promoting Snail1 Crm1-dependent nuclear export 21, 26, 68. These residues follow the characteristic pattern SX_3_SX_3_SX_3_S detected in other GSK3 substrates where phosphorylation takes place gradually from C- to N- Ser/Thr residues 69. Then, when in the cytosol, Snail1 is phosphorylated by CK1ε at Ser104 priming the subsequent phosphorylation at Ser100 and Ser96; this creates a phospho-degron recognized by βTrCP1 that polyubiquitinates and targets Snail1 for proteasomal degradation 26, 68, 70 (Figure 3). This model is also consistent with reports indicating that inhibition of nuclear GSK3β promotes Snail1 stabilization in this compartment. This is accomplished after Wnt stimulation, that promotes the nuclear export of the Axin2/GSK3β complex; therefore, since GSK3β is absent from the nucleus, phosphorylation-induced Snail1 exit to the cytosol is blocked 71.

Although this model of progressive phosphorylation is well-supported by biochemical evidences, several issues need to be clarified. For instance, several results suggest that Ser107 is not phosphorylated by GSK3β. According to the initial model, it would be phosphorylated in the nucleus by GSK3β, as the last temporally modified amino acid in the sequence **S^107^**PAP**S^111^**SFS**S^115^**TSA**S^119^**. However, it is likely that the modification of this Ser is not required for nuclear export and that of serine 111, 115 and 119 is sufficient to uncover the NES and to promote export. In line with this idea, Ser107 phosphorylation by p38 promotes the contrary effect, Snail1 stabilization 72. According to this refinement of the model, modification of this Ser would not be required for nuclear export.

Another point consists in the priming of GSK3β phosphorylation in the nucleus. In contrast to GSK3β action in the cytosol, neither the initiating phosphorylated residue nor the involved protein kinase have been described for the priming of Snail1 phosphorylation in the nucleus by GSK3β. Several data suggest that this might be a consequence of Ser119 phosphorylation by CK2β. The sequence surrounding this Ser (A**S^119^**SLEAE) fits well with the CK2 consensus phosphorylation site 73. Moreover, CK2β phosphorylates Snail1 and enhances further phosphorylation by GSK3β although the precise site has not been identified 74. Accordingly, CK2β negatively controls Snail1 protein expression 74.

Other protein kinases also phosphorylate Snail1 SRD. DYRK2 promotes Ser104 phosphorylation, also priming for the subsequent modification of Ser96 and 100 by GSK3β 75; therefore, it works similarly and alternatively to CK1ε. Interestingly, DYRK2 binding to Snail1 and phosphorylation of Ser104 is prevented by the previous modification of Ser107 by p38, that promotes Snail1 stabilization 72, further suggesting that Ser107 is not involved in Snail1 degradation. It would be interesting to assess if the action of CK1ε on Ser104 is also prevented by Ser107 phosphorylation.

Also present in Snail1 SRD, Ser90 is phosphorylated by G-protein coupled receptor kinase 2 (GRK2) decreasing Snail1 stability 76; however, it is not clear if this modification creates a phospho-degron or promotes Snail1 nuclear export and subsequent degradation.

**Figure 4:**
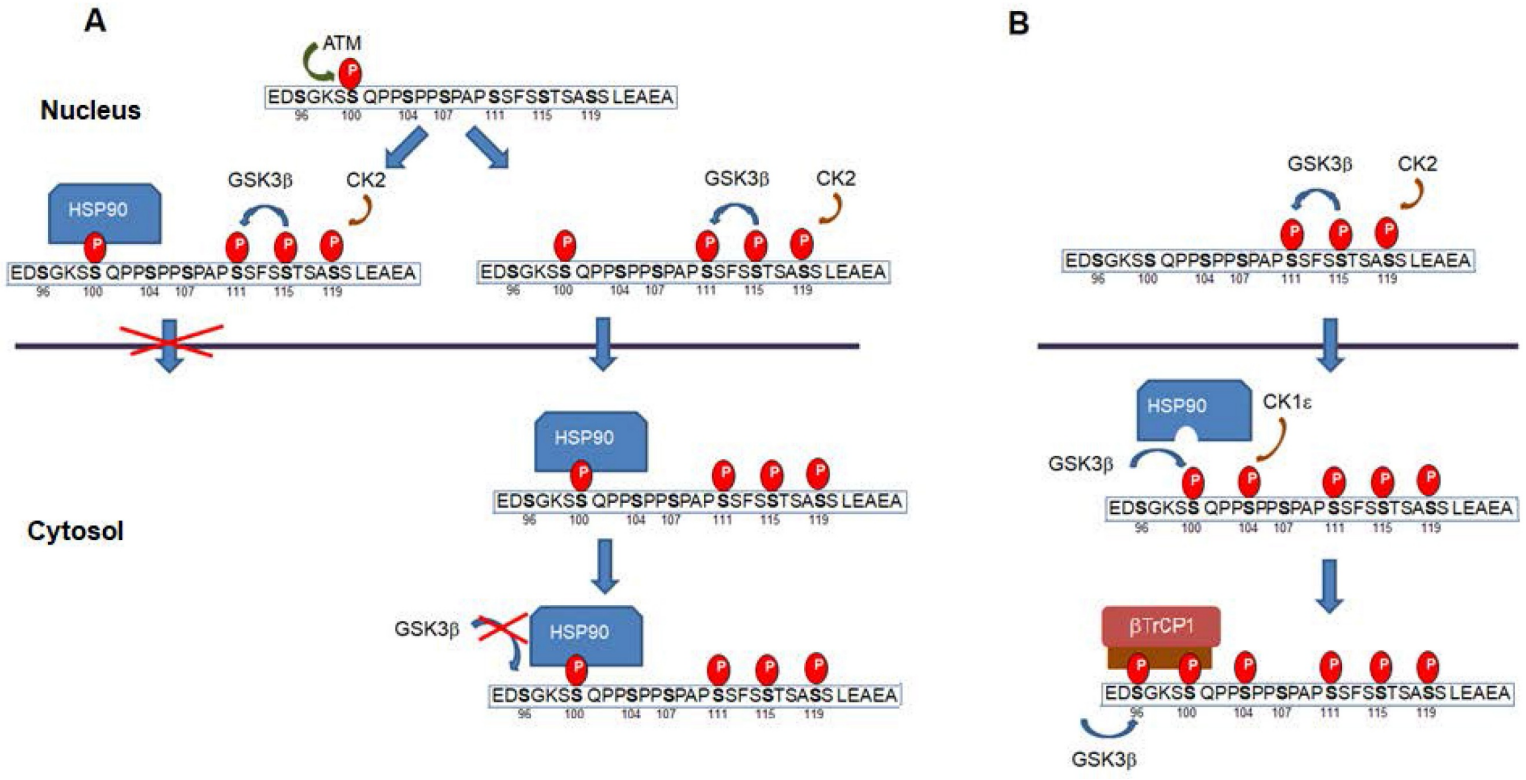
** Snail1 phosphorylation in the nucleus in Ser100 prevents degradation**. (**A**) Snail1 Ser100 phosphorylation in the nucleus by ATM (or DNA-PKc) promotes the recruitment of HSP90 and retention in this compartment. Alternatively, Snail1 phosphorylated in Ser100 might be exported to the cytosol, where is bound by HSP90 preventing further by phosphorylation of Ser96 by GSK3β. However, if Ser104 is modified by CK1ε previous to the modification of Ser100, the association of HSP90 to phosphoSer100 is inhibited (**B**). A similar mechanism might also act when Snail1 is phosphorylated at Ser104 in the nucleus by ERK2. Phosphorylation of this site would facilitate binding by a chaperone and would prevent nuclear exit.

Remarkably, and depending on the context, some of the above-mentioned phosphorylation have a contrary effect since they have been also associated to a higher Snail1 activity (see Figure 2). Both ATM and DNA-dependent protein kinase catalytic subunit (DNA-PKc), two enzymes activated by DNA damage, phosphorylate Snail1 Ser100 promoting Snail1 stability 77-78. This has been related to the Snail1 up-regulation observed after the addition of several agents causing DNA damage. Although Ser100 phosphorylation also participates in the GSK3β-induced Snail1 degradation (see above), these conflicting results have been explained indicating that phosphoSer100 binds HSP90 what stabilizes Snail1 in the nucleus 77. Therefore, Ser100 phosphorylation might produce different effects depending whether it is produced in the cytosol or in the nucleus. At previous discussed, when in the cytosol and catalyzed by GSK3β it would facilitate Ser96 phosphorylation and βTrCP1 dependent degradation; when in the nucleus, it would recruit HSP90, prevent Crm1-mediated nuclear export and stabilize Snail1. A representation of this mechanism is presented in Figure 4a. Alternatively, Snail1 phosphorylated in Ser111-119 and also in Ser100 by ATM might be exported and interact with HSP90 in the cytosol. In any case, binding of HSP90 would prevent phosphorylation by GSK3β in Ser 96 and 100 and the generation of the phosphodegron. This association of HSP90 to phosphoSer100 in the cytosol would be inhibited if the neighbor Ser104 has been previously phosphorylated by CK1ε (Figure 4b).

A similar model also explains Snail1 phosphorylation by ERK2 that takes place in Ser104 79-80 (see Figure 4 legend). As a consequence of its activation by the collagen receptor discoidin domain receptor 2 (DDR2), ERK2 phosphorylates Snail1 in Ser104 and Ser82 and stabilizes it 79. In this case, ERK effects are more complex because this protein kinase modifies an additional site, Ser82 (although this amino acid is not conserved in all mammalian Snail1 proteins) and also decreases GSK3β activity. In any case, it provides an additional example that the phosphorylation of the same residue Ser104 has different effects depending it is produced in the cytosol (by CK1ε) or in the nucleus (by ERK2).

Modification of other sites outside the SRD domain is also relevant for Snail1 function. Phosphorylation of Ser165 (by IKBKE), Thr203 (by Lats or STK39) or Ser246 (by PAK1) increase Snail1 stability and presence in the nucleus 81-84. It has been proposed that these modifications promote Snail1 interaction with unknown nuclear factors preventing the export to the cytosol. The possibility that some of these modifications decrease binding of a specific E3 ubiquitin ligase or enhance the interaction with proteins involved in Snail1 transcriptional activity have not been investigated. Although Ser165, Thr203 and Ser246 are located in the Zn finger domain, Snail1 phosphorylated in these residues is active suggesting that these modifications do not alter Snail1 DNA binding.

Whereas Ser246 increases Snail1 function, modification of Ser249 by the PAR-atypical protein kinase C (aPKC) promotes Snail1 degradation 85. This can be attributed to a preferential cytosolic localization of Ser249-phosphorylated Snail1 since this amino acid is required for the interaction with the importin-β complex, and therefore, its phosphorylation might preclude Snail1 transport to the nucleus 86.

Snail1 is also phosphorylated in Ser11 by protein kinase D1 preventing Snail1 function during EMT 87-88. Several mechanisms have been suggested to explain the inhibitory action of this phosphorylation. First, it has be proposed that it creates a binding site for 14-3-3σ and triggers Snail1 nuclear export 87. It also prevents the interaction with Snail1 co-repressor Ajuba, maintaining Snail1 bound to DNA but inactive 88. Ajuba is a protein essential for the assembly of the Snail1-repressive complex 89. Curiously, Snail1 binding to Ajuba is potentiated by 14-3-3 proteins, although not by 14-3-3σ 90. For other authors, Ser11 phosphorylation generates a phosphodegron, a binding site for FBXO11, that targets Snail1 for proteasomal degradation 27-28. Since these mechanisms do not exclude each other, it is possible that all of them are operative depending on the conditions.

As indicated above, Snail1 stability is regulated through the action of several protein kinases. Some of these kinases and, therefore, Snail1 function are also controlled by different signaling pathways. For instance, Lyn tyrosine kinase, through the stimulation of Vav-Rac1, activates PAK1 that phosphorylates and up-regulates Snail1 protein and function 91. Increased Snail1 function is also the consequence of GSK3 inhibition caused by Akt 26, 92, although GSK3β inhibition and Snail1 stabilization can be produced by alternative mechanisms, such as GSK3b nuclear export (see above, 71) or that involving the hexokinase 2-facilitated phosphorylation of GSK3β by PKA 93.

Also related to the Snail1-induced drug resistance, Snail phosphorylation is controlled by chemotherapeutic drugs. For instance, agents that increase DNA damage, such as camptothecin or ionizing radiation, through the activation of ATM and DNA-PKc phosphorylate Snail1 Ser100 and increase Snail1 stability 77-78. Ionizing radiation has also been reported to inhibit GSK3β, preventing the destabilizing effect of this enzyme on Snail1 94.

Finally, and although many protein kinases have been described to phosphorylate Snail1, few Snail1 phosphatases have been reported so far. Only SCP1 phosphatase dephosphorylates Snail1 and prevents its nuclear export and degradation 60. Although SCP1 has been proposed to dephosphorylate the two sequences phosphorylated by GSK3β in the nucleus and in the cytosol, the preferential localization of this phosphatase in the nucleus suggests that it acts on Ser111, S115 or Ser119, inhibiting nuclear export and therefore, cytosolic degradation. If this phosphatase acts on one of these residues or on all of them remains to be stablished.

As described in this section, Snail1 is extensively phosphorylated by GSK3β controlling its nuclear export and its cytosolic proteasomal degradation. GSK3β action on Snail1 is potentiated by CK1 and CK2 and antagonized by other protein kinases that phosphorylate and stabilize Snail1 in the nucleus. Stabilizing modifications are up-regulated by chemotherapeutic drugs that promote Snail1 expression.

## 4. Other Snail1 post-translational modifications

Among other modifications, acetylation is especially relevant since it controls Snail1 function. Initially described by Yang and coworkers 12, this modification is associated to the switch in Snail1 function, from working as a transcriptional repressor to a transcriptional activator (see 2). Snail1 acetylation is produced by CREB-binding protein (CBP), a protein that interacts with Snail1. The modified residues are Lys146 and 187, placed in the NES and in the first ZnF, respectively 11 (see Figure 1). This might interfere with Snail1 binding to the E-boxes; however, transcriptional activation by Snail1 is not dependent on its direct interaction to DNA; Snail1 binding to activated promoters is indirect, mediated by different co-factors 2. Snail1 acetylation is sensed by bromodomain-containing protein 4 (BRD4), a histone acetylation reader, that interacts with acetylated Snail1 preventing its degradation 95. BRD4 interferes with Snail1 polyubiquitination by βTrCp1 and FBXL14, probably competing with the direct interaction of these E3 ligases 96 and likely also preventing Snail1 nuclear export, since Lys146 is placed very close to the NES. CBP interaction and therefore, Snail1 acetylation is stimulated by Snail1 lactylation 97, although in this case the modified residue has not been identified. It is possible that this Snail1 modification is also catalyzed by CBP, as it has been described in other proteins 97.

Other reports have also confirmed that Snail1 acetylation stabilizes and increases the transcriptional activity of this factor 98-99. For instance, treatment with histone deacetylase inhibitors increases Snail1 through the up-regulation of CSN2, a protein that stabilizes Snail1 61, 98. However, some discrepant results have also been published: Snail1 deacetylation by Sirtuin 1 (SIRT1) has been associated with increased Snail1 function in the nucleus 100. It remains to be established which residues are deacetylated in these conditions. Therefore, it is possible that the relevance of Snail1 acetylation is dependent on the specific function of Snail1 in the different cellular system; thus, if it works preferentially as a transcriptional activator or repressor. For instance, if Snail1 action is predominantly to repress epithelial genes, deacetylation should be required for its action; however, if repression is exerted by other alternative factors (such as Zeb1/2 or Snail2, see 2) and Snail1 works mainly as a transcriptional activator, acetylation should be necessary. At this regard, a Snail1 mutant unable to be acetylated works as transcriptional repressor although does not activate gene expression 11.

Snail1 also undergoes other post-translational modifications that increase its stability and function. For instance, Snail1 interacts with PARP1 and is polyADP-ribosylated by this protein 101-102. The interaction requires the Snail1 C-terminal domain although the precise modified amino acids in Snail1 have not been identified. Snail1 polyADP-ribosylation has been associated to a higher stability and enhanced association to co-repressors such as LSD1, likely because it prevents Snail1 phosphorylation and nuclear export. Snail1 is also sumoylated at Lys234, a modification stimulated by TGFβ and required for invasion 103. Sumoylation increases Snail1 nuclear levels although it is not known if this is produced because it decreases polyubiquitination of Lys234 or enhances the binding to some nuclear factor. Finally, Snail1 is also modified by β-N-acetylglucosamine (O-GlcNAc), a reaction catalyzed by O-GlcNAc transferase and triggered by high-glucose levels 104. This modification has been mapped to Ser112 preventing Snail1 phosphorylation by GSK3β, likely on Ser111 and, therefore stabilizing Snail1 in the nucleus 105. Snail1 glycosylation has also been detected in other conditions that promote Snail1 stabilization 93, 105. Therefore, these modifications seem to prevent Snail1 phosphorylation, both increasing Snail1 presence in the nucleus and stability. Snail1 ADP-ribosylation and glycosylation are promoted by drugs that increase Snail1 expression, such as doxorubicin and oxaliplatin, respectively 102, 105.

Therefore, besides being modified by ubiquitination and phosphorylation, Snail1 undergoes other post-translational modifications. Among these, acetylation is particularly relevant since modulates Snail1 function, from working as a transcriptional repressor to a transcriptional activator. Moreover, other Snail1 modification such as ADP-ribosylation and glycosylation also enhance Snail1 protein stability.

## 5. Insights from Snail1 structure

**Figure 5:**
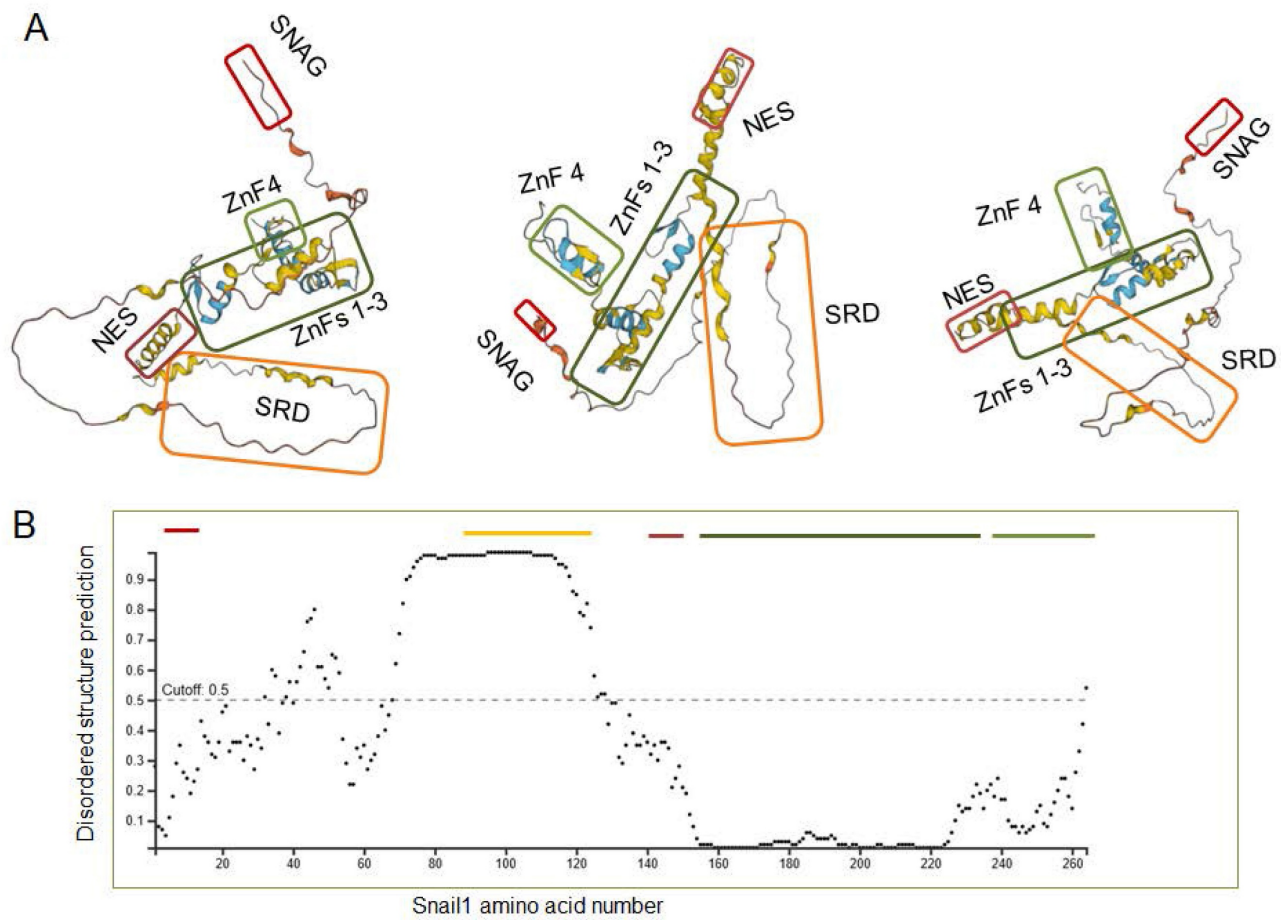
** Predicted Snail1 protein organization**. (**A**) Structures were taken from Alphafold (https://alphafold.ebi.ac.uk/entry/O95863). The different regions of Snail1 protein were highlighted with different boxes: SNAG, red; SRD, orange; NES, brown; ZnF 1-3, dark green; Znf4, light green. Different rotations of the same structure are presented. In **B**, a prediction of disordered regions was assessed with DISOPRED2. The murine Snail1 protein was used in both *in silico* analysis.

A very relevant question consists in why Snail1 is modified by so many enzymes. As presented in Tables 1 and 2, seventeen E3 ligases and twenty-three DUBs have been described to act on Snail1. This indicates that Snail1 protein stability is finely tuned and probably a set of Snail1 E3 ligases and DUBs is specifically expressed in every cell and in every specific condition. This wide number of enzymes might be also related with Snail1 protein organization. Where Snail1 C-terminal domain has been crystalized and is well structured 86, the rest of the protein has been predicted to be poorly organized. Figure 5A shows several representations of the structure predicted by Alphafold (https://alphafold.ebi.ac.uk/entry/O95863) 106-107 where the different elements of the N -and C-terminal Snail1 domains have been highlighted. As seen, this program suggests that Snail1 protein is arranged as a core formed by the Zn fingers 1-3 with other protruding elements, all of these showing very little regular secondary structure. The proposed protein organization fits well with the experimental data; for instance, Ser111, Ser115 and Ser119, whose phosphorylation regulates nuclear export, are predicted to be relatively close to the NES (amino acids 132-144), suggesting that their modification might facilitate NES accessibility to Crm1.

It has also been predicted that the most of N-terminal part, particularly the SRD is intrinsically disordered 108. This is also confirmed by an analysis with DISOPRED2 tool 109 as shown in Figure 5B. This might be relevant for Snail1 function, as discussed below, but it might be also the cause of artifacts. For instance, the number of DUBs acting on Snail1 seems excessive; it is possible that some of them might regulate Snail1 in a more indirect fashion; for instance, controlling the activity of factors required for Snail1 expression, and its interaction with Snail1, particularly when it has been determined by coimmunoprecipitation of overexpressed proteins, is artifactual.

Intrinsically disordered regions (IDRs) are especially abundant in nuclear proteins, particularly in transcription factors 110. IDRs are defined by the existence of several conformational ensembles what has suggested that proteins with IDRs might have the capability to associate to a high number of factors 111. However, despite this variable structure, many IDRs display selectivity 112. In Snail1, the presence of IDR in most of the N-terminal regulatory domain might be required for its role as an interaction hub, regulating the assembly of the transcriptional activation complex and increasing the local concentration of members of this complex. Therefore, during the transcription of mesenchymal genes Snail1 would act as a specialized scaffold protein.

## 6. Conclusions, open questions and future perspectives

In general terms, as seen above and on the basis of the alterations in Snail1 function, Snail1 post-translational modifications are classified in three large groups: A) modifications that regulate Snail1 protein stability. These are the polyubiquitination of several Lys that target Snail1 to the proteasome and the phosphorylation of Ser in the SRD, that promote binding of βTrCP1 and other E3 ubiquitin ligases. Other modifications also impact Snail1 stability; for instance, Lys63-mediated polyubiquitination or sumoylation prevent Snail1 Lys48-polyubiquitination and degradation by the proteasome. B) Modifications that control Snail1 presence in the nucleus. These consist mainly in the phosphorylation of different residues in the SRD and other domains, that either facilitate the access of Crm1 to the NES and enhance nuclear export or, on the contrary, promote the interaction of Snail1 with nuclear proteins and prevent the export. Other alterations such as glycosylation might also stimulate nuclear retention inhibiting the phosphorylation of specific residues involved in export. C) Modifications that directly affect Snail1 function, preventing Snail1 binding to the DNA or regulating its association with co-repressors, such as Ajuba or with co-activators, as the GATA zinc finger protein p66β 113. Few modifications are classified in this category. Only Snail1 Ser11 phosphorylation by PKD decreases Snail1 binding to Ajuba and therefore, Snail1 repression of transcription 88. However, the most relevant is acetylation that is associated to an increased Snail1 binding to co-activators. In any case, acetylation also stimulates Snail1 protein stability, as previously commented 95. Regarding this modification, biochemical *in vitro* assays need to be performed to assess if acetylation indeed modifies the direct association of Snail1 to DNA, to co-repressors or co-activators. A possibility to be considered is that acetylation is required for stabilizing Snail1 interaction with CBP itself, being this protein the main responsible for the effect of Snail1 on transcriptional activation.

As discussed above, some modifications might have a different role if they are produced in the cytosol or in the nucleus, or if they are accompanied by other modifications. This is the case for the phosphorylation of Ser100 and Ser104 that, when performed by ATM and ERK2 in the nucleus have a positive effect on Snail1 stability, whereas when catalyzed by GSK3β and CK1ε in the cytosol participate in Snail1 degradation (see Figure 4). An explanation for these results has been provided in section 3. As indicated, the phosphorylation promoted by ATM or ERK2 might facilitate the interaction with some nuclear structures and inhibit Crm1 binding and nuclear export. It is unlikely that these activating phosphorylations by themselves decrease the interaction with Crm1, since all the modified residues are in sequences far from the NES (Figure 2); however, they might promote a more compact structure that would be less accessible to CK2 and GSK3β, the protein kinases that phosphorylate Snail1 SRD and induce Snail1 nuclear export.

Many different biochemical issues on Snail1 structure and on the impact of post-translational modifications need to be investigated. Some of them are presented Table 3. For instance, the relevance of Snail1 acetylation in Snail1 binding to E-boxes, to SNAG-interacting co-repressors or to co-activators, or the identification of factors specifically bound by phosphorylated residues when these modifications increase Snail1 stability.

Another issue of interest consists in the identification of drugs promoting Snail1 degradation. So far, no specific inhibitors of Snail1 DUBs have been characterized. It seems unlikely that compounds targeting Snail1 DUBs in tumor cells might have a therapeutic use, considering the high number of Snail1 DUBs that have been described and their different expression in many cancer cells. It would be more interesting to inhibit Snail1 DUBs in tumor-activated fibroblast, since Snail1 is also crucial for the pro-invasive function of these cells 114, that are less heterogeneous than epithelial tumor cells. Moreover, so far only USP29X has been shown to control Snail1 stability in these cells 13. Another feasible line of research might consist in the identification of compounds increasing the expression of Snail1 E3 ligases such as βTrCP1 and FBXL14, the most active ones. Alternatively, drugs enhancing Snail1 interaction with these E3 ligases might be also very useful. At this respect, Snail1 degradation is boosted by metformin, a drug that reverses EMT 115. This compound enhances Snail1 interaction with FBXL14, facilitating Snail1 degradation 37. It is possible that compounds working similarly to metformin might decrease Snail1 expression and increase the cytotoxic action of DNA damaging agents.

In any case, the variety and extent of Snail1 post-translational modifications highlight the importance of Snail1 protein stabilization in the control of EMT and the cellular properties associated to this transition, such tumor invasion and chemoresistance.

## Figures and Tables

**Table 1:** **E3 ubiquitin ligases interacting with Snail1 and controlling Snail1 proteasomal degradation**. The reported Snail1 E3 ubiquitin ligases are presented indicating if they de-stabilize (-) or stabilize (+) Snail1. Only enzymes that have been demonstrated to interact with Snail1 by co-immunoprecipitation are shown. They are presented in the chronological order they were reported.

Snail1 E3 ubiquitin ligase	Effect on Snail1 stability	Reference
FBXW1 (βTrCP1)	-	26
FBXL14	-	9
FBXL5	-	25
FBXO11	-	27, 29
FBXO45	-	39
FBXO31	-	34
FBXW7	-	30, 31
FBXO22	-	35
PPIL2	-	116
SPSB3	-	36
TRIM21	-	40
HECTD1	-	117
TRIM50	-	42
MARCH2	-	118
FBXL8	-	119
Peli1 (Pellino1)	+	48
TNFAIP3 (A20 E3 ligase)	+	49

**Table 2:** **Deubiquitinases interacting with Snail1 and controlling Snail1 proteasomal degradation**. Only enzymes that have been demonstrated to interact with Snail1 by co-immunoprecipitation are shown. They are presented in the chronological order they were reported.

Snail1 deubiquitinase	Reference
USP17L2 (DUB3)	12, 50
USP47	51
PSMD14	120
OTUB1	121
USP27X	13
USP1	57
USP3	122
USP26	123
USP11	124
USP37	125, 126
USP18	127
USP29	58, 59
EIF3H	128
USP9X	129
USP36	52
USP10	130, 131
OTUD4	132, 133
USP41	134
USP28	135
USP22	136
USP30	137
USP35	138
USP5	66

**Table 3:** **Open questions on the regulation of Snail1 function.** See more details in the text.

Snail1 modification	Open questions
Polyubiquitination	Is the action of different Snail1 E3 ligases coordinated?
	How many Snail1 E3 ligases recognize a phospho-degron?
	How relevant is Snail1 degradation by the lysosome in tumors?
	How many Snail1 E3 ligases and DUBs increase or eliminate polyubiquitination in in vitro assays?
	Which factors interact with monoubiquitinated Snail1 promoting its stabilization?
	
Phosphorylation	Does CK2 phosphorylate Snail1 Ser119 and prime further Ser115 phosphorylation by GSK3β?
	Does Hsp90 binding to phosphorylated Ser100 prevent Ser96 phosphorylation by GSK3β?
	Which nuclear factors interact with phosphorylated Ser100 or Ser104?
	Do phosphorylation of Ser165, Thr203 or Ser246 preclude Snail1 nuclear export by binding to specific nuclear factors?
	Does Snail1 Ser11 phosphorylation modulate interaction of co-repressors with the SNAG box in in vitro assays?
	
Acetylation	Does acetylation prevent Snail1 interaction with E boxes or affect SNAG binding co-repressors?
	Does acetylation facilitate Snail1 interaction with transcriptional co-activators?
	Does acetylation have an impact on Snail1 protein stability
	
Other	Does Snail parylation, glycosylation or lactylation avoid Snail1 phosphorylation and nuclear export?
	

## References

[1] Yang J, Antin P, Berx G, Blanpain C, Brabletz T, Bronner M (2020). Guidelines and definitions for research on epithelial-mesenchymal transition. Nat Rev Mol Cell Biol.

[2] Garcia de Herreros A (2024). Dual role of Snail1 as transcriptional repressor and activator. Biochim Biophys Acta Rev Cancer.

[3] Baulida J, Díaz VM, Garcia de Herreros A (2019). Snail1: A Transcriptional Factor Controlled at Multiple Levels. J Clin Med.

[4] Lu Z, Ghosh S, Wang Z, Hunter T (2003). Downregulation of caveolin-1 function by EGF leads to the loss of E-cadherin, increased transcriptional activity of beta-catenin, and enhanced tumor cell invasion. Cancer Cell.

[5] Peinado H, Quintanilla M, Cano A (2003). Transforming growth factor beta-1 induces snail transcription factor in epithelial cell lines: mechanisms for epithelial mesenchymal transitions. J Biol Chem.

[6] Rosanò L, Spinella F, Di Castro V, Nicotra MR, Dedhar S, García de Herreros A (2005). Endothelin-1 promotes epithelial-to-mesenchymal transition in human ovarian cancer cells. Cancer Res.

[7] Grotegut S, von Schweinitz D, Christofori G, Lehembre F (2006). Hepatocyte growth factor induces cell scattering through MAPK/Egr-1-mediated upregulation of Snail. EMBO J.

[8] Hudson LG, Choi C, Newkirk KM, Parkhani J, Cooper KL, Lu P (2007). Ultraviolet radiation stimulates expression of Snail family transcription factors in keratinocytes. Mol Carcinog.

[9] Viñas-Castells R, Beltran M, Valls G, Gómez I, García JM, Montserrat-Sentís B (2010). The hypoxia-controlled FBXL14 ubiquitin ligase targets SNAIL1 for proteasome degradation. J Biol Chem.

[10] Sun M, Song L, Zhou T, Gillespie GY, Jope RS (2011). The role of DDX3 in regulating Snail. Biochim Biophys Acta.

[11] Hsu DS, Wang HJ, Tai SK, Chou CH, Hsieh CH, Chiu PH (2014). Acetylation of snail modulates the cytokinome of cancer cells to enhance the recruitment of macrophages. Cancer Cell.

[12] Wu Y, Wang Y, Lin Y, Liu Y, Wang Y, Jia J (2017). Dub3 inhibition suppresses breast cancer invasion and metastasis by promoting Snail1 degradation. Nat Commun.

[13] Lambies G, Miceli M, Martínez-Guillamon C, Olivera-Salguero R, Peña R, Frías C (2019). TGFβ-Activated USP27X Deubiquitinase Regulates Cell Migration and Chemoresistance via Stabilization of Snail1. Cancer Res.

[14] Fuertes G, Del Valle-Pérez B, Pastor J, Andrades E, Peña R, García de Herreros A (2023). Noncanonical Wnt signaling promotes colon tumor growth, chemoresistance and tumor fibroblast activation. EMBO Rep.

[15] Tang X, Sui X, Weng L, Liu Y (2021). SNAIL1: Linking Tumor Metastasis to Immune Evasion. Front Immunol.

[16] Khan AQ, Hasan A, Mir SS, Rashid K, Uddin S, Steinhoff M (2024). Exploiting transcription factors to target EMT and cancer stem cells for tumor modulation and therapy. Semin Cancer Biol.

[17] Villarejo A, Cortés-Cabrera A, Molina-Ortíz P, Portillo F, Cano A (2014). Differential role of Snail1 and Snail2 zinc fingers in E-cadherin repression and epithelial to mesenchymal transition. J Biol Chem.

[18] Matthews JM, Kowalski K, Liew CK, Sharpe BK, Fox AH, Crossley M A class of zinc fingers involved in protein-protein interactions biophysical characterization of CCHC fingers from fog and U-shaped. Eur J Biochem. 200; 267: 1030-8.

[19] Yamasaki H, Sekimoto T, Ohkubo T, Douchi T, Nagata Y, Ozawa M (2005). Zinc finger domain of Snail functions as a nuclear localization signal for importin beta-mediated nuclear import pathway. Genes Cells.

[20] Mingot JM, Vega S, Maestro B, Sanz JM, Nieto MA (2009). Characterization of Snail nuclear import pathways as representatives of C2H2 zinc finger transcription factors. J Cell Sci.

[21] Domínguez D, Montserrat-Sentís B, Virgós-Soler A, Guaita S, Grueso J, Porta M (2003). Phosphorylation regulates the subcellular location and activity of the snail transcriptional repressor. Mol Cell Biol.

[22] Molina-Ortiz P, Villarejo A, MacPherson M, Santos V, Montes A, Souchelnytskyi S (2012). Characterization of the SNAG and SLUG domains of Snail2 in the repression of E-cadherin and EMT induction: modulation by serine 4 phosphorylation. PLoS One.

[23] Liu X, Pan YJ, Kang MJ, Jiang X, Guo ZY, Pei DS (2023). PAK5 potentiates slug transactivation of N-cadherin to facilitate metastasis of renal cell carcinoma. Cell Signal.

[24] Díaz VM, García de Herreros A (2016). F-box proteins: Keeping the epithelial-to-mesenchymal transition (EMT) in check. Semin Cancer Biol.

[25] Viñas-Castells R, Frías Á, Robles-Lanuza E, Zhang K, Longmore GD, García de Herreros A (2014). Nuclear ubiquitination by FBXL5 modulates Snail1 DNA binding and stability. Nucleic Acids Res.

[26] Zhou BP, Deng J, Xia W, Xu J, Li YM, Gunduz M (2004). Dual regulation of Snail by GSK-3beta-mediated phosphorylation in control of epithelial-mesenchymal transition. Nat Cell Biol.

[27] Zheng H, Shen M, Zha YL, Li W, Wei Y, Blanco MA (2014). PKD1 Phosphorylation-Dependent Degradation of SNAIL by SCF-FBXO11 Regulates Epithelial-Mesenchymal Transition and Metastasis. Cancer Cell.

[28] Li M, Zhang L, Guan T, Huang L, Zhu Y, Wen Y (2024). Energy Stress-activated AMPK Phosphorylates Snail1 and Suppresses its Stability and Oncogenic Function. Cancer Lett.

[29] Jin Y, Shenoy AK, Doernberg S, Chen H, Luo H, Shen H (2015). FBXO11 promotes ubiquitination of the Snail family of transcription factors in cancer progression and epidermal development. Cancer Lett.

[30] Xiao G, Li Y, Wang M, Li X, Qin S, Sun X (2018). FBXW7 suppresses epithelial-mesenchymal transition and chemo-resistance of non-small-cell lung cancer cells by targeting snai1 for ubiquitin-dependent degradation. Cell Prolif.

[31] Zhang Y, Zhang X, Ye M, Jing P, Xiong J, Han Z (2018). FBW7 loss promotes epithelial-to-mesenchymal transition in non-small cell lung cancer through the stabilization of Snail protein. Cancer Lett.

[32] Davis RJ, Markus W, Clurman BE (2006). Tumor Suppression by the Fbw7 Ubiquitin Ligase: Mechanisms and Opportunities. Nat Rev Immunol.

[33] Li N, Babaei-Jadidi R, Lorenzi F, Spencer-Dene B, Clarke P, Domingo E (2019). An FBXW7-ZEB2 axis links EMT and tumour microenvironment to promote colorectal cancer stem cells and chemoresistance. Oncogenesis.

[34] Zou S, Ma C, Yang F, Xu X, Jia J, Liu Z (2017). FBXO31 Suppresses Gastric Cancer EMT by Targeting Snail1 for Proteasomal Degradation. Mol Cancer Res.

[35] Sun R, Xie HY, Qian JX, Huang YN, Yang F, Zhang FL (2018). FBXO22 possesses both protumorigenic and antimetastatic roles in breast cancer progression. Cancer Res.

[36] Liu Y, Zhou H, Zhu R, Ding F, Li Y, Cao X (2018). SPSB3 targets SNAIL for degradation in GSK-3β phosphorylation-dependent manner and regulates metastasis. Oncogene.

[37] Song L, Guo J, Chang R, Peng X, Li J, Xu X (2018). LKB1 obliterates Snail stability and inhibits pancreatic cancer metastasis in response to metformin treatment. Cancer Sci.

[38] Lander R, Nordin K, LaBonne C (2011). The F-box protein Ppa is a common regulator of core EMT factors Twist, Snail, Slug, and Sip1. J. Cell Biol.

[39] Xu M, Zhu C, Zhao X, Chen C, Zhang H, Yuan H (2015). Atypical ubiquitin E3 ligase complex Skp1-Pam-Fbxo45 controls the core epithelial-to-mesenchymal transition-inducing transcription factors. Oncotarget.

[40] Jin Y, Zhang Y, Li B, Zhang J, Dong Z, Hu X (2019). TRIM21 mediates ubiquitination of Snail and modulates epithelial to mesenchymal transition in breast cancer cells. Int J Biol Macromol.

[41] Grassi G, Di Caprio G, Santangelo L, Fimia GM, Cozzolino AM, Komatsu M (2015). Autophagy regulates hepatocyte identity and epithelial-to-mesenchymal and mesenchymal-to-epithelial transitions promoting Snail degradation. Cell Death Dis.

[42] Li R, Zhu L, Peng Y, Zhang X, Dai C, Liu D (2021). TRIM50 Suppresses Pancreatic Cancer Progression and Reverses the Epithelial-Mesenchymal Transition via Facilitating the Ubiquitous Degradation of Snail1. Front Oncol.

[43] Di Rienzo M, Romagnoli A, Antonioli M, Piacentini M, Fimia GM (2020). TRIM proteins in autophagy: selective sensors in cell damage and innate immune responses. Cell Death Differ.

[44] Fusco C, Mandriani B, Di Rienzo M, Micale L, Malerba N, Cocciadiferro D (2018). TRIM50 regulates Beclin 1 proautophagic activity. Biochim Biophys Acta Mol Cell Res.

[45] Kimura T, Jain A, Choi SW, Mandell MA, Schroder K, Johansen T (2015). TRIM-mediated precision autophagy targets cytoplasmic regulators of innate immunity. J Cell Biol.

[46] Ye WL, Huang L, Yang XQ, Wan S, Gan WJ, Yang Y (2024). (2024) TRIM21 induces selective autophagic degradation of c-Myc and sensitizes regorafenib therapy in colorectal cancer. Proc Natl Acad Sci USA.

[47] Ryu KJ, Lee KW, Park SH, Kim T, Hong KS, Kim H (2024). Chaperone-mediated autophagy modulates Snail1 protein stability: implications for breast cancer metastasis. Mol Cancer.

[48] Jeon YK, Kim CK, Hwang KR, Park HY, Koh J, Chung DH (2017). Pellino-1 promotes lung carcinogenesis via the stabilization of Slug and Snail through K63-mediated polyubiquitination. Cell Death Differ.

[49] Lee JH, Jung SM, Yang KM, Bae E, Ahn SG, Park JS (2017). A20 promotes metastasis of aggressive basal-like breast cancers through multi-monoubiquitylation of Snail1. Nat Cell Biol.

[50] Liu T, Yu J, Deng M, Yin Y, Zhang H, Luo K (2017). CDK4/6-dependent activation of DUB3 regulates cancer metastasis through SNAIL1. Nat Commun.

[51] Choi BJ, Park SA, Lee SY, Cha YN, Surh YJ (2017). Hypoxia induces epithelial-mesenchymal transition in colorectal cancer cells through ubiquitin-specific protease 47-mediated stabilization of Snail: A potential role of Sox9. Sci Rep.

[52] Qin K, Yu S, Liu Y, Guo R, Guo S, Fei J (2023). USP36 stabilizes nucleolar Snail1 to promote ribosome biogenesis and cancer cell survival upon ribotoxic stress. Nat Commun.

[53] Prakash V, Carson BB, Feenstra JM, Dass RA, Sekyrova P, Hoshino A (2019). Ribosome biogenesis during cell cycle arrest fuels EMT in development and disease. Nat Commun.

[54] Lin Y, Wang Y, Shi Q, Yu Q, Liu C, Feng J (2017). Stabilization of the transcription factors slug and twist by the deubiquitinase dub3 is a key requirement for tumor metastasis. Oncotarget.

[55] Ouchida AT, Kacal M, Zheng A, Ambroise G, Zhang B, Norberg E (2018). USP10 regulates the stability of the EMT-transcription factor Slug/SNAI2. Biochem Biophys Res Commun.

[56] Qiu W, Cai X, Xu K, Song S, Xiao Z, Hou Y (2022). PRL1 Promotes Glioblastoma Invasion and Tumorigenesis via Activating USP36-Mediated Snail2 Deubiquitination. Front Oncol.

[57] Sonego M, Pellarin I, Costa A, Vinciguerra GLR, Coan M, Kraut A (2019). USP1 links platinum resistance to cancer cell dissemination by regulating Snail stability. Sci Adv.

[58] Wu Y, Zhang Y, Wang D, Zhang Y, Zhang J, Zhang Y (2020). USP29 enhances chemotherapy-induced stemness in non-small cell lung cancer via stabilizing Snail1 in response to oxidative stress. Cell Death Dis.

[59] Qian W, Li Q, Wu X, Li W, Li Q, Zhang J (2020). Deubiquitinase USP29 promotes gastric cancer cell migration by cooperating with phosphatase SCP1 to stabilize Snail protein. Oncogene.

[60] Wu Y, Evers BM, Zhou BP (2009). Small C-terminal domain phosphatase enhances snail activity through dephosphorylation. J Biol Chem.

[61] Wu Y, Deng J, Rychahou PG, Qiu S, Evers BM, Zhou BP (2009). Stabilization of Snail by NF-κB Is Required for Inflammation-Induced Cell Migration and Invasion. Cancer Cell.

[62] Yang J, Liao Y, Wang B, Cui L, Yu X, Wu F (2023). EDARADD promotes colon cancer progression by suppressing E3 ligase Trim21-mediated ubiquitination and degradation of Snail. Cancer Lett.

[63] Yang X, Li J, Zeng W, Li C, Mao B (2016). Elongator Protein 3 (Elp3) stabilizes Snail1 and regulates neural crest migration in Xenopus. Sci Rep.

[64] Jang D, Kwon H, Choi M, Lee J, Pak Y (2019). Sumoylation of Flotillin-1 promotes EMT in metastatic prostate cancer by suppressing Snail degradation. Oncogene.

[65] Kim SH, Ryu KJ, Hong KS, Kim H, Han H, Kim M (2024). ERK3 Increases Snail Protein Stability by Inhibiting FBXO11-Mediated Snail Ubiquitination. Cancers.

[66] Hong KS, Ryu KJ, Kim H, Kim M, Park SH, Kim T (2025). MSK1 promotes colorectal cancer metastasis by increasing Snail protein stability through USP5-mediated Snail deubiquitination. Exp Mol Med.

[67] Zhang Y, Zuo D, Qiu J, Li K, Niu Y, Yuan Y (2022). NXN suppresses metastasis of hepatocellular carcinoma by promoting degradation of Snail through binding to DUB3. Cell Death Dis.

[68] Yook JI, Li XY, Ota I, Fearon ER, Weiss SJ (2005). Wnt-dependent regulation of the E-cadherin repressor snail. J Biol Chem.

[69] Doble BW, Woodgett JR (2003). GSK-3: tricks of the trade for a multi-tasking kinase. J Cell Sci.

[70] Xu Y, Lee SH, Kim HS, Kim NH, Piao S, Park SH (2010). Role of CK1 in GSK3Β-mediated phosphorylation and degradation of Snail. Oncogene.

[71] Yook JI, Li XY, Ota I, Hu C, Kim HS, Kim NH (2006). A Wnt-Axin2-GSK3beta cascade regulates Snail1 activity in breast cancer cells. Nat Cell Biol.

[72] Ryu KJ, Park SM, Park SH, Kim IK, Han H, Kim HJ (2019). p38 Stabilizes Snail by Suppressing DYRK2-Mediated Phosphorylation That Is Required for GSK3β-βTrCP-Induced Snail Degradation. Cancer Res.

[73] Meggio F, Marin O, Pinna LA (1994). Substrate specificity of protein kinase CK2. Cell Mol Biol Res.

[74] Deshiere A, Duchemin-Pelletier E, Spreux E, Ciais D, Combes F, Vandenbrouck Y (2013). Unbalanced expression of CK2 kinase subunits is sufficient to drive epithelial-to-mesenchymal transition by Snail1 induction. Oncogene.

[75] Mimoto R, Taira N, Takahashi H, Yamaguchi T, Okabe M, Uchida K (2013). DYRK2 controls the epithelial-mesenchymal transition in breast cancer by degrading Snail. Cancer Lett.

[76] Palacios-García J, Sanz-Flores M, Asensio A, Alvarado R, Rojo-Berciano S, Stamatakis K (2020). G-protein-coupled receptor kinase 2 safeguards epithelial phenotype in head and neck squamous cell carcinomas. Int J Cancer.

[77] Sun M, Guo X, Qian X, Wang H, Yang C, Brinkman KL (2012). Activation of the ATM-Snail pathway promotes breast cancer metastasis. J Mol Cell Biol.

[78] Pyun BJ, Seo HR, Lee HJ, Jin YB, Kim EJ, Kim NH (2013). Mutual regulation between DNA-PKcs and snail1 leads to increased genomic instability and aggressive tumor characteristics. Cell Death Dis.

[79] Zhang K, Corsa CA, Ponik SM, Prior JL, Piwnica-Worms D, Eliceiri KW (2013). The collagen receptor discoidin domain receptor 2 stabilizes SNAIL1 to facilitate breast cancer metastasis. Nat Cell Biol.

[80] Xie B, Lin W, Ye J, Wang X, Zhang B, Xiong S (2015). DDR2 facilitates hepatocellular carcinoma invasion and metastasis via activating ERK signaling and stabilizing SNAIL1. J Exp Clin Cancer Res.

[81] Yang Z, Rayala S, Nguyen D, Vadlamudi RK, Chen S, Kumar R (2005). Pak1 phosphorylation of Snail, a master regulator of epithelial-to- mesenchyme transition, modulates Snail's subcellular localization and functions. Cancer Res.

[82] Zhang K, Rodriguez-Aznar E, Yabuta N, Owen RJ, Mingot JM, Nojima H (2012). Lats2 kinase potentiates Snail1 activity by promoting nuclear retention upon phosphorylation. EMBO J.

[83] Xie W, Jiang Q, Wu X, Wang L, Gao B, Sun Z (2022). IKBKE phosphorylates and stabilizes Snail to promote breast cancer invasion and metastasis. Cell Death Differ.

[84] Qiu Z, Dong B, Guo W, Piotr R, Longmore G, Yang X (2021). STK39 promotes breast cancer invasion and metastasis by increasing SNAI1 activity upon phosphorylation. Theranostics.

[85] Jung HY, Fattet L, Tsai JH, Kajimoto T, Chang Q, Newton AC (2019). Apical-basal polarity inhibits epithelial-mesenchymal transition and tumour metastasis by PAR-complex-mediated SNAI1 degradation. Nat Cell Biol.

[86] Choi S, Yamashita E, Yasuhara N, Song J, Son SY, Won YH (2014). Structural basis for the selective nuclear import of the C2H2 zinc-finger protein Snail by importin β. Acta Crystallogr D Biol Crystallogr.

[87] Du C, Zhang C, Hassan S, Biswas MHU, Balaji KC (2010). Protein kinase D1 suppresses epithelial-to-mesenchymal transition through phosphorylation of snail. Cancer Res.

[88] Bastea LI, Döppler H, Balogun B, Storz P (2012). Protein kinase D1 maintains the epithelial phenotype by inducing a DNA-bound, inactive SNAI1 transcriptional repressor complex. PLoS One.

[89] Ayyanathan K, Peng H, Hou Z, Fredericks WJ, Goyal RK, Langer EM (2007). The Ajuba LIM domain protein is a corepressor for SNAG domain mediated repression and participates in nucleocytoplasmic shuttling. Cancer Res.

[90] Hou Z, Peng H, White DE, Wang P, Lieberman PM, Halazonetis T (2010). 14-3-3 binding sites in the snail protein are essential for snail-mediated transcriptional repression and epithelial-mesenchymal differentiation. Cancer Res.

[91] Thaper D, Vahid S, Nip KM, Moskalev I, Shan X, Frees S (2017). Targeting Lyn regulates Snail family shuttling and inhibits metastasis. Oncogene.

[92] Katoh M, Katoh M (2006). Cross-talk of WNT and FGF signaling pathways at GSK3beta to regulate beta-catenin and SNAIL signaling cascades. Cancer Biol Ther.

[93] Blaha CS, Ramakrishnan G, Jeon SM, Nogueira V, Rho H, Kang S (2022). A non-catalytic scaffolding activity of hexokinase 2 contributes to EMT and metastasis. Nat Commun.

[94] Nagarajan D, Melo T, Deng Z, Almeida C, Zhao W (2012). ERK/GSK3beta/Snail signaling mediates radiation-induced alveolar epithelial-to-mesenchymal transition. Free Radic Biol Med.

[95] Qin ZY, Wang T, Su S, Shen LT, Zhu GX, Liu Q (2019). BRD4 Promotes Gastric Cancer Progression and Metastasis through Acetylation-Dependent Stabilization of Snail. Cancer Res.

[96] Fan M, Yang K, Wang X, Chen L, Gill PS, Ha T (2023). Lactate promotes endothelial-to-mesenchymal transition via Snail1 lactylation after myocardial infarction. Sci Adv.

[97] Chen Y, Wu J, Zhai L, Zhang T, Yin H, Gao H (2024). Metabolic regulation of homologous recombination repair by MRE11 lactylation. Cell.

[98] Xu W, Liu H, Liu ZG, Wang HS, Zhang F, Wang H (2018). Histone deacetylase inhibitors upregulate Snail via Smad2/3 phosphorylation and stabilization of Snail to promote metastasis of hepatoma cells. Cancer Lett.

[99] Han JH, Kim YK, Kim H, Lee J, Oh MJ, Kim SB (2022). Snail acetylation by autophagy-derived acetyl-coenzyme A promotes invasion and metastasis of KRAS-LKB1 co-mutated lung cancer cells. Cancer Commun.

[100] Lee YH, Kim SJ, Fang X, Song NY, Kim DH, Suh J (2022). JNK-mediated Ser27 phosphorylation and stabilization of SIRT1 promote growth and progression of colon cancer through deacetylation-dependent activation of Snail. Mol Oncol.

[101] Rodríguez MI, González-Flores A, Dantzer F, Collard J, García de Herreros A, Oliver FJ (2011). Poly(ADP-ribose)-dependent regulation of Snail1 protein stability. Oncogene.

[102] Lin Y, Kang T, Zhou BP (2014). Doxorubicin enhances Snail/LSD1-mediated PTEN suppression in a PARP1-dependent manner. Cell Cycle.

[103] Gudey SK, Sundar R, Heldin CH, Bergh A, Landström M (2017). Pro-invasive properties of Snail1 are regulated by sumoylation in response to TGFβ stimulation in cancer. Oncotarget.

[104] Park SY, Kim HS, Kim NH, Ji S, Cha SY, Kang JG (2010). Snail1 is stabilized by O-GlcNAc modification in hyperglycaemic condition. EMBO J.

[105] Hua Q, Lu Y, Wang D, Da J, Peng W, Sun G (2023). KIAA1199 promotes oxaliplatin resistance and epithelial mesenchymal transition of colorectal cancer via protein O-GlcNAcylation. Transl Oncol.

[106] Jumper J, Evans R, Pritzel A, Green T, Figurnov M, Ronneberger O (2021). Highly accurate protein structure prediction with AlphaFold. Nature.

[107] Varadi M, Bertoni D, Magana P, Paramval U, Pidruchna I, Radhakrishnan M (2024). AlphaFold Protein Structure Database in 2024: providing structure coverage for over 214 million protein sequences. Nucleic Acids Res.

[108] Mooney SM, Jolly MK, Levine H, Kulkarni P (2016). Phenotypic plasticity in prostate cancer: role of intrinsically disordered proteins. Asian J Androl.

[109] Fan X, Kurgan LA (2014). Accurate prediction of disorder in protein chains with a comprehensive and empirically designed consensus. J Biomol Struct Dyn.

[110] Frege T, Uversky VN (2015). Intrinsically disordered proteins in the nucleus of human cells. Biochem Biophys Rep.

[111] Piovesan D, Necci M, Escobedo N, Monzon AM, Hatos A, Mičetić I (2021). MobiDB: intrinsically disordered proteins in 2021. Nucleic Acids Res.

[112] Cermakova K, Hodges HC (2023). Interaction modules that impart specificity to disordered protein. Trends Biochem Sci.

[113] Zou X, Ma L, Zhang Y, Zhang Q, Xu C, Zhang D (2023). GATA zinc finger protein p66β promotes breast cancer cell migration by acting as a co-activator of Snail. Cell Death Dis.

[114] Alba-Castellón L, Olivera-Salguero R, Mestre-Farrera A, Peña R, Herrera M, Bonilla F (2016). Snail1-Dependent Activation of Cancer-Associated Fibroblast Controls Epithelial Tumor Cell Invasion and Metastasis. Cancer Res.

[115] Chen YC, Li H, Wang J (2020). Mechanisms of metformin inhibiting cancer invasion and migration. Am J Transl Res.

[116] Jia Z, Wang M, Li S, Li X, Bai XY, Xu Z (2018). U-box ubiquitin ligase PPIL2 suppresses breast cancer invasion and metastasis by altering cell morphology and promoting SNAI1 ubiquitination and degradation. Cell Death Dis.

[117] Wang X, De Geyter C, Jia Z, Peng Y, Zhang H (2020). HECTD1 regulates the expression of SNAIL: Implications for epithelial-mesenchymal transition. Int J Oncol.

[118] Ito K, Harada I, Martinez C, Sato K, Lee E, Port E (2024). MARCH2, a Novel Oncogene-regulated SNAIL E3 Ligase, Suppresses Triple-negative Breast Cancer Metastases. Cancer Res Commun.

[119] Li Y, Zuo C, Wu X, Ding Y, Wei Y, Chen S (2024). FBXL8 inhibits post-myocardial infarction cardiac fibrosis by targeting Snail1 for ubiquitin-proteasome degradation. Cell Death Dis.

[120] Zhu R, Liu Y, Zhou H, Li L, Li Y, Ding F (2018). Deubiquitinating enzyme PSMD14 promotes tumor metastasis through stabilizing SNAIL in human esophageal squamous cell carcinoma. Cancer Lett.

[121] Zhou H, Liu Y, Zhu R, Ding F, Cao X, Lin D (2018). OTUB1 promotes esophageal squamous cell carcinoma metastasis through modulating Snail stability. Oncogene.

[122] Fan L, Chen Z, Wu X, Cai X, Feng S, Lu J (2019). Ubiquitin-Specific Protease 3 Promotes Glioblastoma Cell Invasion and Epithelial-Mesenchymal Transition via Stabilizing Snail. Mol Cancer Res.

[123] Li L, Zhou H, Zhu R, Liu Z (2019). USP26 promotes esophageal squamous cell carcinoma metastasis through stabilizing Snail. Cancer Lett.

[124] Wang W, Wang J, Yan H, Zhang K, Liu Y (2019). Upregulation of USP11 promotes epithelial-to-mesenchymal transition by deubiquitinating Snail in ovarian cancer. Oncol Rep.

[125] Xiao Z, Chang L, Kim J, Zhang P, Hang Q, Yap S (2029). USP37 is a SNAI1 deubiquitinase. Am J Cancer Res.

[126] Cai J, Li M, Wang X, Li L, Li Q, Hou Z (2020). USP37 Promotes Lung Cancer Cell Migration by Stabilizing Snail Protein via Deubiquitination. Front Genet.

[127] Huang F, Zheng C, Huang L, Lin C, Wang J (2020). USP18 directly regulates Snail1 protein through ubiquitination pathway in colorectal cancer. Cancer Cell Int.

[128] Guo X, Zhu R, Luo A, Zhou H, Ding F, Yang H (2020). EIF3H promotes aggressiveness of esophageal squamous cell carcinoma by modulating Snail stability. J Exp Clin Cancer Res.

[129] Guan T, Yang X, Liang H, Chen J, Chen Y, Zhu Y (2022). Deubiquitinating enzyme USP9X regulates metastasis and chemoresistance in triple-negative breast cancer by stabilizing Snail1. J Cell Physiol.

[130] Luo Y, Zhu Q, Xiang S, Wang Q, Li J, Chen X (2023). Downregulated circPOKE promotes breast cancer metastasis through activation of the USP10-Snail axis. Oncogene.

[131] Zhu W, Ye B, Yang S, Li Y (2023). USP10 promotes intrahepatic cholangiocarcinoma cell survival and stemness via SNAI1 deubiquitination. J Mol Histol.

[132] Gao Y, Tang J, Ma X, Zhang C, Huang L, Che J (2023). OTUD4 regulates metastasis and chemoresistance in melanoma by stabilizing Snail1. J Cell Physiol.

[133] Ma X, Wan R, Wen Y, Liu T, Song Y, Zhu Y (2024). Deubiquitinating enzyme OTUD4 regulates metastasis in triple-negative breast cancer by stabilizing Snail1. Exp Cell Res.

[134] Yoon JY, Seo SU, Woo SM, Kwon TK (2023). USP41 Enhances Epithelial-Mesenchymal Transition of Breast Cancer Cells through Snail Stabilization. Int J Mol Sci.

[135] Liu Z, Yu Y, Zhou S, Zhang X, Zhou Z (2023). Inhibition of Pard3 promotes breast cancer metastasis via the USP28 mediated deubiquitination of Snail1. Heliyon.

[136] Wei C, Zhao X, Zhang H, Wang L (2023). USP2 promotes cell proliferation and metastasis in choroidal melanoma via stabilizing Snail. J Cancer Res Clin Oncol.

[137] Sun K, Liao S, Yao X, Yao F (2024). USP30 promotes the progression of breast cancer by stabilising Snail. Cancer Gene Ther.

[138] Ma C, Tian Z, Wang D, Gao W, Qian L, Zang Y (2024). Ubiquitin-specific Protease 35 Promotes Gastric Cancer Metastasis by Increasing the Stability of Snail1. Int J Biol Sci.

